# New Horizons in Myopia Management: Bridging Epidemiology and Clinical Innovation

**DOI:** 10.3390/vision8040068

**Published:** 2024-12-01

**Authors:** Nir Erdinest, Yair Morad

**Affiliations:** 1 Department of Ophthalmology, Hadassah-Hebrew University Medical Center, Faculty of Medicine, Hebrew University of Jerusalem, Jerusalem 91120, Israel; 2 Shamir Medical Center, Department of Ophthalmology, Tel Aviv University, Zerifin 70300, Israel; yair.morad@gmail.com

In 1975, Brit J. published an editorial letter titled “Changing views on myopia”, offering a snapshot of the prevailing theories of the time [1]. Research on myopia was still in its infancy, and there was limited understanding of the underlying mechanisms and treatment options. Johannes Kepler’s proof that myopia was caused by parallel rays of light focusing in front of the retina back in 1604 was a cornerstone [2], birthing the belief that a longer axial length was the primary cause of myopia [1]. In the present day, the field of myopia research and treatment has undergone substantial evolution. It has become one of the most extensively studied areas within ophthalmology, with an exponential trend in the volume of literature published on this topic indicative of a continuous increase in the rate of the research output (Figure 1).

These data were collected using the search query “myopia” and only including articles with “Myopia” as part of their title (“myopia [Title]”).

We now find ourselves in an era of unprecedented progress in the management of myopia, with a plethora of research focusing on interventions to slow down its progression, particularly in children and teenagers. In addition, genetic studies, imaging, and attempts to identify its molecular mechanisms are underway. The paper by Brennan et al. [3] argues that despite the numerous publications on myopia management, a consensus is lacking on what “efficacy” means in this context. They highlight the challenges in comparing the efficacy of different treatments, indicating that axial elongation should be the preferred endpoint for assessing myopic progression according to the historical rationale. Moreover, their paper emphasizes that the available data on the efficacy of myopia treatment are often limited to those from short-term studies spanning two to three years. This raises questions about the long-term effects of these interventions, emphasizing the need for research of a more extended duration to understand their long-term impact further. We want to emphasize the significant increase in the prevalence of myopia in childhood observed in recent decades and acknowledge a broader call for a more proactive approach to myopia management. Given the limited interventions available and that the current data indicate more significant myopia progression in younger people, some contend that treatment should be implemented for all young myopes [3,4]. Our collective works advocate for an in-depth understanding of the risk factors associated with myopia, which is essential for formulating effective preventive measures and treatment plans.

We highlight the urgent need for additional research to understand the role of the restricted outdoor exposure and early academic pressures of the modern world as potential risk factors for myopia [5,6,7]. COVID-19-pandemic-related lockdowns may have exacerbated these issues due to increased screen time and reduced outdoor activities. Indeed, in some cases, decreased effectiveness of atropine treatment has been observed [8].

Public health interventions have become increasingly crucial in tackling the widespread prevalence of myopia. Targeted prevention strategies are essential, including education of health providers and the public, especially in high-risk areas [5,6]. The International Myopia Institute has been instrumental in the evolvement of a common language and a unified set of goals for researchers and clinicians. However, the field is still fraught with conflicting data [9].

Myopia is a multifactorial condition impacted by genetics, lifestyle, and environmental variables, which complicates prediction. The current calculations rely on peer-reviewed research, commercial software calculators, online risk assessment tools, and mobile health apps. These allow practitioners and the general public to gauge their risk levels. Experimental artificial intelligence (AI) and machine learning models are being developed to offer potentially more accurate predictions [10,11,12]. These technologies show promise in addressing the complexities of myopia management, from diagnosis to monitoring. AI underscores the efficacy of the current algorithms, particularly in detecting pathological myopia, which could potentially ease clinical workloads in the future [10,12,13]. The next phase in myopia management after detection can include pharmacological treatments, lifestyle changes, and potentially gene therapy.

This Special Issue, “New Horizons in Myopia Management: Bridging Epidemiology and Clinical Innovation”, focuses on myopia and related visual disorders, covering its prevalence, treatment methods, and early detection to prevent irreversible visual impairment. This collection comprises three publications [Contributions 1–3].

While myopia management in childhood remains a critical concern among eye care professionals in India, a recent survey by Naik et al. [Contribution 1] reveals notable gaps in awareness and clinical practice in this area. Their survey of Indian optometrists explored current practices and perspectives on myopia management in childhood [Contribution 1]. Concerningly, this study revealed a lack of awareness among the respondents regarding the ocular complications associated with high myopia, with a substantial share of them unable to identify key risks. Despite the growing knowledge in terms of emerging strategies for myopia management, such as orthokeratology and multifocal contact lenses, their adoption rate remains low, with single-vision distance spectacles still predominantly being prescribed. This discrepancy between awareness and clinical practice is suggestive of potential barriers to implementing the optimal myopia care, with this study identifying medicolegal concerns, the absence of clear clinical practice guidelines, and inadequate consultation times.

Furthermore, this study found that optometrists primarily rely on their personal clinical experience and research articles, rather than established guidelines or continuing professional development programs, when acquiring information. This reliance on potentially less robust sources of information underscores the need for greater emphasis on evidence-based practice and the development of accessible, comprehensive guidelines for myopia management in India. This study’s findings emphasize the urgent need for targeted interventions, such as continuing education programs and the establishment of clear clinical guidelines, to improve the quality of myopia management in childhood and reduce the long-term risks associated with this increasingly prevalent condition [Contribution 1].

While Naik et al. [Contribution 1] underscore the need for enhanced guidelines and training in myopia management, some of the other research in this Special Issue advances our understanding of the potential mechanisms underlying effective interventions. Amorim-de-Sousa et al. investigated the physiological effects of extended depth-of-focus (EDOF) lenses, designed to manage myopia, on the retinal responses of young adults [Contribution 2]. Using multifocal electroretinography to compare the retinal electrical activity elicited by EDOF lenses and standard single-vision lenses, these researchers revealed a significant enhancement in the inner foveal response when using the former. This localized effect suggests a potential mechanism according to which these lenses may control myopia. The authors hypothesize that the EDOF lens design, which incorporates negative spherical aberration and induces peripheral defocus, may contribute to this observed change in retinal activity. However, the precise interplay between these optical factors and the retinal response remains to be fully understood. Despite this study providing compelling evidence on the retinal mechanism involved in controlling myopia using EDOF lenses, further investigation is necessary to confirm this hypothesis and explore the long-term implications of these retinal changes [Contribution 2].

These studies highlight the importance of standardized clinical practices and accurate measurement techniques. Building on tools for precise monitoring, Rizzieri and Facchin evaluated the reliability of two key methods for axial length measurement, offering insights into the choice of instrumentation in studies of myopia progression [Contribution 3]. Their study examined the agreement between axial length measurements obtained using partial coherence interferometry and spectral-domain optical coherence tomography in adults with varying refractive errors, finding a high level of agreement between the two methods, with a negligible and statistically insignificant mean difference. However, a Bland–Altman analysis revealed a small but statistically significant proportional bias, indicating that the difference between the measurements was slightly greater in eyes with longer or shorter axial lengths. Despite its small magnitude, this proportional bias suggests that caution is warranted when interchanging between PCI and SD-OCT in studies of longitudinal myopia progression, as small disparities could accumulate over time and potentially confound the interpretation of the treatment efficacy. This study underscores the importance of maintaining consistent instrumentation to accurately monitor changes in axial length in myopia management, particularly in studies evaluating the effectiveness of interventions that aim to delay axial elongation [Contribution 3].

This collection of studies advances our understanding of myopia management by exploring diverse perspectives. It examines the challenges in clinical practice and gaps in awareness, investigates treatment mechanisms involving specialized lenses, and highlights the significance of accurate measurement methods. These findings underscore the essential need for standardized practices, enhanced education, and precise monitoring tools within myopia control.

The future of research on myopia hinges on evidence-based clinical management and technological advances. Clinicians often grapple with conflicting data, underscoring the need for public health campaigns and digital education platforms. International collaboration, as is facilitated by organizations like the International Myopia Institute, is crucial given the global scope of this issue.

In summary, tackling myopia demands a unified effort from clinicians, researchers, and policy-makers. We must focus on evidence-based prevention, public education, and global cooperation in addressing this public health crisis.

## Figures and Tables

**Figure 1:**
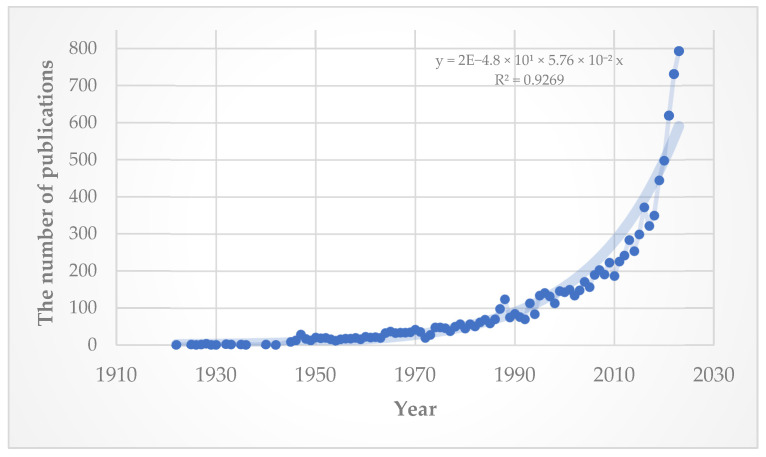
Century-long trends in publication counts for articles sourced from the PubMed/MEDLINE^®^ search engine from 1922 to 2023.
